# Relevance and Therapeutic Possibility of PTEN-Long in Renal Cell Carcinoma

**DOI:** 10.1371/journal.pone.0114250

**Published:** 2015-02-25

**Authors:** Hui Wang, Peng Zhang, Chunhua Lin, Qingxia Yu, Jitao Wu, Lin Wang, Yupeng Cui, Ke Wang, Zhenli Gao, Hong Li

**Affiliations:** 1 Department of Urology, the Affiliated Yantai Yuhuangding Hospital of Qingdao University Medical College, Institute of Urology, Zhifu, Yantai, Shandong, 264000, People’s Republic of China; 2 Critical Care Medicine, the Affiliated Yantai Yuhuangding Hospital of Qingdao University Medical College, Zhifu, Yantai, Shandong, 264000, People’s Republic of China; 3 Minimally Invasive Abdominal Surgery, Ningbo Medical Center, LiHuiLi hospital, Ningbo, Zhejiang, 315040, People’s Republic of China; University of Medicine, Greifswald, Germany, Germany

## Abstract

PTEN-Long is a translational variant of PTEN (Phosphatase and Tensin Homolog). Like PTEN, PTEN-Long is able to antagonize the PI3K-Akt pathway and inhibits tumor growth. In this study, we investigated the role PTEN-Long plays in the development and progression of clear cell renal cell carcinoma (ccRCC) and explored the therapeutic possibility using proteinaceous PTEN-Long to treat ccRCC. We found that the protein levels of PTEN-Long were drastically reduced in ccRCC, which was correlated with increased levels of phosphorylated Akt (pAkt). Gain of function experiments showed overexpression of PTEN-Long in the ccRCC cell line 786-0 suppressed PI3K-Akt signaling, inhibited cell proliferation, migration and invasion, and eventually induced cell death. When purified PTEN-Long was added into cultured 786-0 cells, it entered cells, blocked Akt activation, and induced apoptosis involving Caspase 3 cleavage. Furthermore, PTEN-Long inhibited proliferation of 786-0 cells in xenograft mouse model. Our results implicated that understanding the roles of PTEN-Long in renal cell carcinogenesis has therapeutic significance.

## Introduction

Renal cell carcinoma (RCC) is one of the most common cancers with high degree of malignancy in urinary system. RCC originates in the urinary tubule system, also known as renal adenocarcinoma, accounting for 80–90% of kidney cancers. In China, RCC is the second largest urogenital tumor after bladder cancer, accounting for 2–3% of adult malignancies and 20% of pediatric malignancies. There are significant differences in the incidence rates in terms of sex with male to female ratio of 2∶1. The incidence of RCC increases with age with high prevalence in patients at 40 to 55 years of age. Because the cause of RCC remains largely unknown [1] and the incidence of the death rate from kidney cancer in China has risen during recent years [2], RCC represents a major therapeutic challenge.

Among signaling pathways proposed to be involved in RCC, PTEN/phosphoinositide 3-kinase (PI3K)/Akt pathway has been well studied [3]–[7]. Akt, a serine/threonine protein kinase, has been demonstrated to control the balance between survival and apoptosis [8]. Akt activation likely plays a crucial role in the carcinogenesis and progression in RCC, as it has been observed to have an elevated activity in human malignant tumors, including RCC. The activation of Akt has been shown to inversely correlate with the expression of PTEN. In many cancers, the tumor suppressor PTEN is often lost or mutated [9], [10]. Alterations in PTEN expression may predispose RCC formation [3], [5], [7], [11]. The phosphatase encoded by PTEN has dual-specificity, and its primary substrate is phosphatidylinositol 3, 4, 5 triphosphate (PIP3) [12]–[16]. PTEN antagonizes PI3K through its lipid phosphatase activity, which affects many downstream cellular activities including growth, proliferation, and survival [17]–[20]. It has been shown that PTEN’s tumor-suppressing abilities are not only limited to tumor cells. Deletion of PTEN from tumor microenvironment also results in neoplastic growth [21]–[23]. Recently, a translational variant of PTEN, PTEN-Long, has been identified by Parsons and colleagues that has a similar function to PTEN [24]–[26]. PTEN-Long varies from PTEN by having an in-frame alternative translation initiation codon upstream of the canonical ATG in the PTEN transcript. PTEN-Long, like PTEN, suppresses tumor growth through antagonizing the PI3K pathway and reducing PI3K signaling in a phosphatase-dependent manner. When xenograft breast tumor models were treated with PTEN-Long, tumor regression was induced.

In this study we analyzed protein levels of PTEN and PTEN-Long and Akt phosphorylation in a cohort of 50 ccRCC and corresponding normal tissues. We found reduction or loss of PTEN-Long expression was reversely correlated with levels of pAkt in ccRCC. In order to dissect the contribution of PTEN and PTEN-long to ccRCC, we investigated effects of PTEN or PTEN-long in PTEN-null clear cell renal cancer cell line 786-0. Our results show that unlike PTEN, PTEN-Long when added in cultured cells, as a purified protein, inhibited intracellular signaling through its lipid phosphatase activity and induced apoptosis, indicating PTEN-Long is able to work as proteinaceous therapeutics.

## Materials and Methods

### Preparation of protein extract and protein purification

The present study was approved by Yantai Yuhuangding Hospital Ethics Committee with an approval number of 2011–103. Written informed consent for the use of their tissues in this study was obtained from all participants involved. For preparation of protein extracts, renal tumor tissue and normal renal tissue were crushed with a mortar under liquid nitrogen and suspended on ice in lysis buffer (20 mM Hepes, pH 7.7, 0.2 M NaCl, 1.5 mM MgCl2, 0.4 mM EDTA, 1% Triton X-100, 0.5 mM DTT, 2 mM phenylmethylsulphonyl fluoride, 20 mM β-glycerophosphate and 0.1 mM sodium-ortho-vanadate) containing protease inhibitor cocktail (Roche, Basel, Switzerland). Protein concentrations of the lysates were determined using Bradford Protein Assay from Bio-Rad. Recombinant PTEN-Long and related proteins were purified as described previously [24].

### Western blotting

Whole-cell extracts were prepared in radioimmunoprecipitation assay buffer (RIPA) (50 mM Tris [pH 8.0], 150 mM NaCl, 0.5% deoxycholate, 0.1% SDS and 1.0% NP-40) (Thermo Scientific, Rockford, IL, USA) containing a protease inhibitor cocktail. Total protein (50 µg per well) was separated by sodium dodecyl sulfate-polyacrylamide gel electrophoresis (SDS-PAGE) and transferred onto polyvinylidene fluoride membranes (PVDF, Immobilon P, Millipore, Bedford, MA) for Western blotting. Western blotting was carried out using standard protocols. In brief, the membrane was blocked in 5% low fat milk powder in PBST (3.2 mM Na_2_HPO_4_, 0.5 mM KH_2_PO_4_, 1.3 mM KCl, 135 mM NaCl, 0.05% Tween-20, pH 7.4) for 30 min. The primary antibodies against indicated proteins were incubated overnight at 4°C. After wash, the membrane was incubated with horseradish peroxidase (HRP)-conjugated secondary antibodies (Santa Cruz Biotechnology) for 30 min at room temperature. The bound antibodies were visualized by an enhanced chemiluminescence (ECL) detection system. For quantification, Western blots were imaged using Image J software (NIH). Antibody against PTEN was purchased from CASCADE BIOSCIENCE (Winchester, MA, USA), Akt, pAkt, Cleaved Caspase 3, PRAS40, and pPRAS40 were from Cell Signaling (Danvers, MA, USA), whereas Actin was from Santa Cruz Biotechnology (Santa Cruz, CA, USA).

### Cell culture

Human clear cell renal carcinoma 786-0 cell was obtained from the American Type Culture Collection (ATCC, Manassas, VA, USA). Cells were cultured as a monolayer in RPMI-1640 medium supplemented with 10% of fetal bovine serum (FBS), 50 mg/ml penicillin and 50 mg/ml streptomycin (Invitrogen, Carlsbad, CA, USA). Cells were maintained in an incubator with a humidified atmosphere of 95% and 5% CO_2_ at 37°C.

### Cell transfection

786-0 cells were transiently transfected with indicated constructs using Lipofectamin 2000 transfection reagent (Life Technology, Carlsbad, CA, USA) according to the manufacturer’s protocol. In brief, cells were seeded in 100-mm dishes (Nunc, Roskilde, Denmark) 24 h before transfection and transiently transfected at a confluency of 80–90%. Mock transfection without plasmids was used as a control. The transfection mixture was diluted in Opti-MEM serum-free media (Life Technology, Carlsbad, CA, USA) and cells were incubated in Opti-MEM serum-free media as well. 6 h after transfection, the transfection media was changed to the complete media containing 10% FBS and antibiotics. Cells were harvested 48 h after transfection for protein extraction.

### Immunoprecipitation

Immunoprecipitations were performed essentially as described earlier [27]. In brief, cells were lysed as described above. Lysate was incubated with 3 µl of anti-PTEN antibodies bound to Protein A/G beads (GE Healthcare Life Sciences, Pittsburg, PA, USA) by gentle rocking at 4°C overnight. The beads were washed three times with lysis buffer and one time with PBS. Bound proteins were eluted by the addition of 2X Laemmli buffer, resolved on SDS-PAGE gels, and analyzed by immunoblotting.

### Proliferation and apoptosis assay

For proliferation assays, 0.2 million cells stably expressing indicated proteins were seeded, cells were harvested daily, and cell numbers were counted, in triplicates. For apoptosis assay, cells expressing different proteins were grown to 60% confluence in complete media before switching to serum free media. Cells were then harvested after 24 h, 48 h, and 72 h respectively and stained with Annexin V-FITC (Clontech, Mountain View, CA, USA) according to the manufacturer’s protocol. Stained cells were immediately analyzed by flow cytometry. For PTEN-Long treatment in culture, cells were washed once with serum free media and incubated in serum free media containing purified PTEN-Long or related proteins at different doses. Cells were treated for different times and then harvested for Western blotting analysis for cleaved caspase 3 or subjected to imaging analysis or evaluated by propidium iodide (PI) staining/flow for percentages of sub G_0_/G_1_ population.

### Migration and invasion assays

Cell migration into a wound area was performed as previously described [28] with slight modifications. In brief, cells expressing indicated proteins were grown to subconfluency on culture dishes. The cells were starved of serum for 24 h and treated with Mitomycin C for 90 min to arrest cell proliferation. A wound track was then introduced by scraping the cell monolayer with a thin pipette tip. Cells were then analyzed with phase-contrast microscope for up to 12 h. A cytoselect 24-well cell invasion assay kit (Cell Biolabs, Inc., San Diego, CA) was used for invasion assays according to the manufacturer’s protocol.

### Xenograft assays and ethics statement

Xenograft assays were performed in accordance with Institutional Animal Care and Use Committee guidelines. The protocol was approved by the Committee of the Ethics of Animal Experiments of the Yantai Yuhuangding Hospital of Qingdao University Medical College (Permit Number: 2011211). All surgery was performed under isoflurane anesthesia, and all efforts were made to minimize suffering. In brief, 786-0 cells (1.5×10^6^) were implanted subcutaneously into the flank of athymic nude mice and allowed to grow until the tumor reached ∼0.5 cm in diameter. Mice carrying tumors were then randomly assigned into two groups and were injected intraperitoneally with purified PTEN or PTEN-Long at 5 mg/kg daily. Tumor size was measured every other day by a single observer. After final measurement on day 10, mice were euthanized with carbon dioxide inhalation. Mice were exposed to 100% carbon dioxide at 5 PSI for a minimum of 3 minutes in a cage as recommended in institutional Euthanasia Guidelines for Investigators. The mice were left undisturbed for an additional 15 minutes. Prior to disposal, death was confirmed by palpating for the absence of an apex heart beat and a lack of respiration.

### Statistical analysis

Descriptive statistics were used to summarize study data. Quantitative (Mann-Whitney U test) comparisons were performed using nonparametric assays. Statistical significance was defined as a two-tailed *P*≤0.05. All statistical analyses were performed using a commercially available statistical software package (SPSS, Chicago, IL, or SAS, Durham, NC).

## Results

### PTEN-Long inversely correlates pAkt in ccRCC

**Figure 1:**
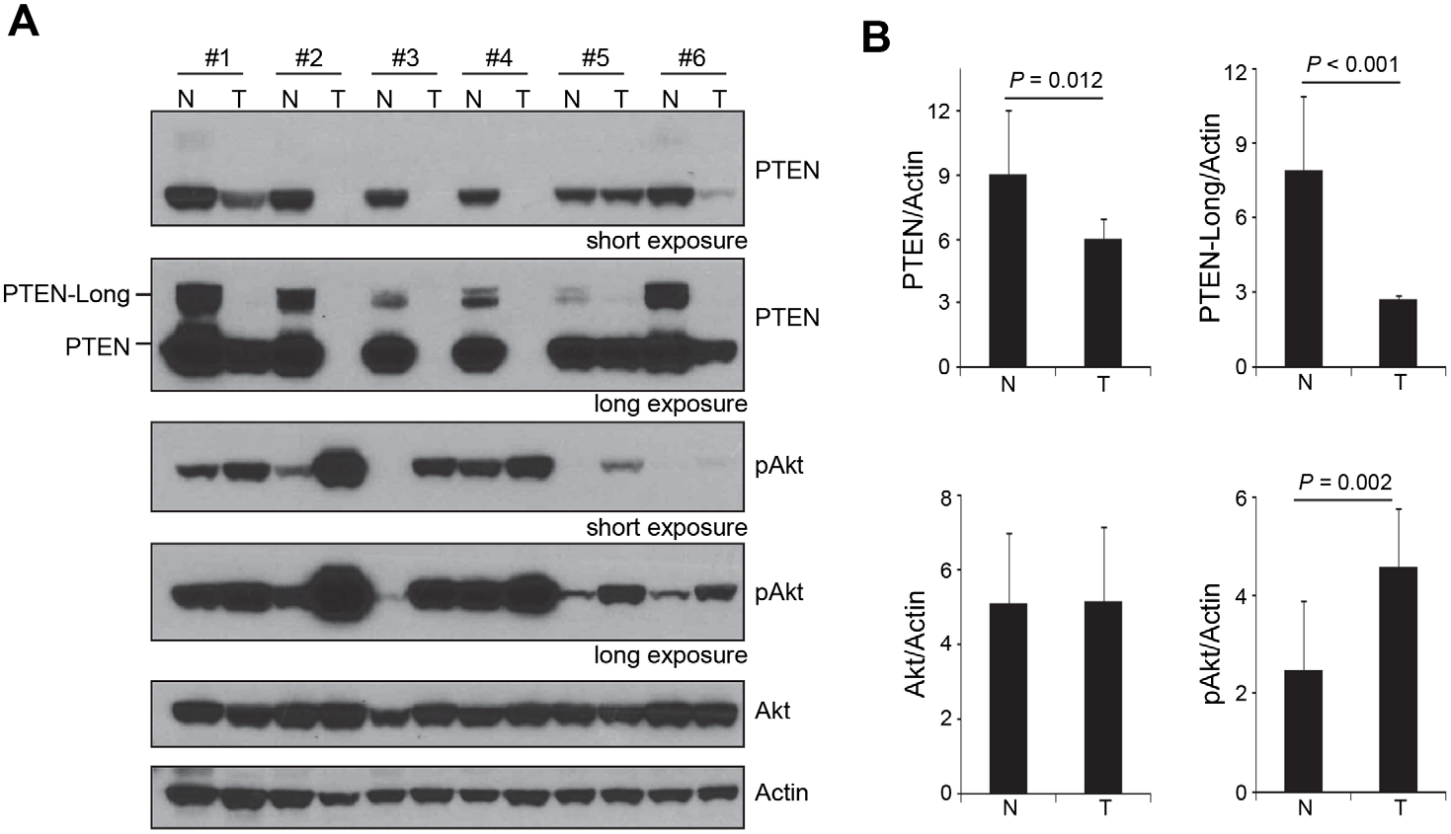
Protein expression of PTEN-Long was significantly reduced or lost in ccRCC. **A**, Western blot analysis of PTEN, PTEN-Long, and pAkt. Represented are 6 pairs of clear cell renal cell carcinomas and their corresponding normal renal tissues. In clear cell renal cell carcinomas the PTEN-Long expression was either negative or very weak, compared to normal renal tissue. N, normal renal tissue; T, tumor tissue. **B**, The ratio of PTEN, PTEN-Long, Akt, and pAkt to Actin in 45 samples. The PTEN-Long expression in ccRCC was reduced significantly in comparison with the corresponding normal renal tissue. Note that tumor samples with undetectable PTEN-Long and PTEN were excluded from quantification.

To study if PTEN-Long plays a role in ccRCC, we examined protein expression of PTEN-Long in 50 ccRCC and the corresponding normal renal tissue of the same patients using Western blotting analysis. PTEN-Long was expressed in all investigated normal renal tissue specimens. However, PTEN-Long expression in ccRCCs was markedly reduced in comparison with normal tissue, as evidenced by Western blotting analysis (Fig. 1A). In 35% of all ccRCCs studied, PTEN-Long expression was undetectable, probably due to genomic loss of PTEN or truncation mutations that destabilize PTEN or PTEN-Long proteins. Akt is well known to be activated by phosphorylation at Ser 473. We therefore examined the Akt activation status in ccRCC by western blotting using antibody against phosphorylated Akt (pAkt), which recognizes only phosphorylated Akt at Ser 473. The expression of total Akt protein in ccRCC and the corresponding kidney tissue were at similar levels. In contrast, the levels of pAkt were observed to increase significantly in ccRCC, in comparison with levels of pAkt in the corresponding normal kidney tissue (Fig. 1A). Therefore, the pAkt level inversely correlated with the PTEN-Long level.

To quantify the expression of PTEN-Long in ccRCC, the levels of PTEN-Long in renal tumor tissue were measured by computer-aided integration of the bands and then compared with the levels in corresponding normal renal tissue. For each tumor, the ratio of PTEN-Long expression to Actin in both renal tumor tissue and the corresponding normal renal tissue was calculated. We found there is a striking reduction of PTEN-Long expression in ccRCCs versus normal tissue. While the average level of PTEN in ccRCC was about 65% of that in corresponding normal renal tissue, the average level of PTEN-Long was less than 35% (*P*<0.001) of that in corresponding normal renal tissue (Fig. 1B). Consistent with PTEN and PTEN-Long’s lipid phosphatase activity, pAkt in ccRCC studied increased to almost two-fold in ccRCC, in comparison with corresponding normal renal tissue (Fig. 1B).

### Overexpression of PTEN-Long suppressed of PI3K-Akt signaling in 786-0 ccRCC cells

**Figure 2:**
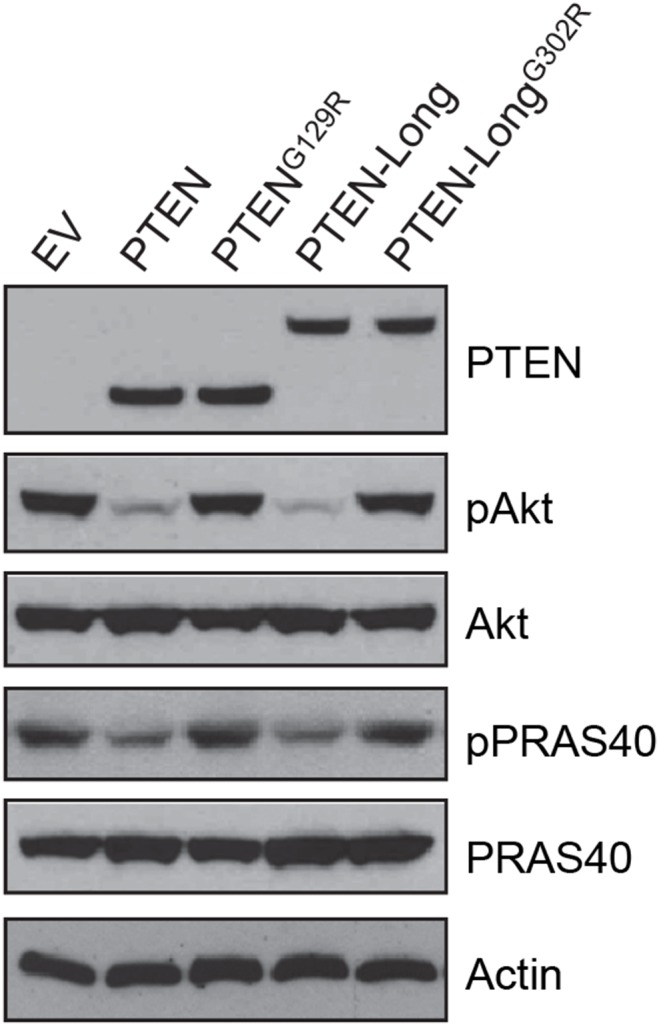
Overexpression of PTEN-Long suppressed PI3K-Akt signaling in 786-0 cells. Western blot analysis of lysates from 786-0 cells showing overexpression of PTEN or PTEN-Long, but not PTEN^G129R^ or PTEN-Long^G302R^, suppressed Akt phosphorylation (Ser473) and its downstream event.

To characterize the role of PTEN-Long in kidney cancer cells and the mechanisms of tumorigenesis, we stably overexpressed PTEN-Long in 786-0 cells that lacks the PTEN and PTEN-Long gene. In order to avoid co-expression of PTEN-Long and PTEN from PTEN-Long cDNA, we mutated the 5′ initiation site from CTG to ATG and the canonical initiation site from ATG to ATA in *PTEN-Long* [24]. Cell lysates were then subjected to PTEN, pAkt (Ser473), and Akt immunoblotting analysis. As shown in Fig. 2, the basal levels of Akt phosphorylation were reduced by roughly 90% in PTEN and PTEN-Long expressing cells, compared to empty vector (EV) transfected cells. This indicates PTEN-Long has similar lipid phosphatase activity as PTEN and is able to inhibit PI3K signaling in the cells in which it was expressed. A missense mutation in the phosphatase domain of PTEN-Long (G302R)–analogous to G129R in PTEN, a tumor mutation with depleted phosphatase activity–failed to inhibit Akt phosphorylation (Fig. 2, lanes 1, 4, and 5). Together, the effects of overexpression of equivalent amounts of PTEN, PTEN-Long, or their respective mutated analogs in 786-0 cells confirmed that like PTEN, PTEN-Long is able to suppress PI3K-Akt signaling pathway in a lipid phosphatase-dependent manner.

### Overexpression of PTEN-Long inhibits cell proliferation and induces cell death

**Figure 3:**
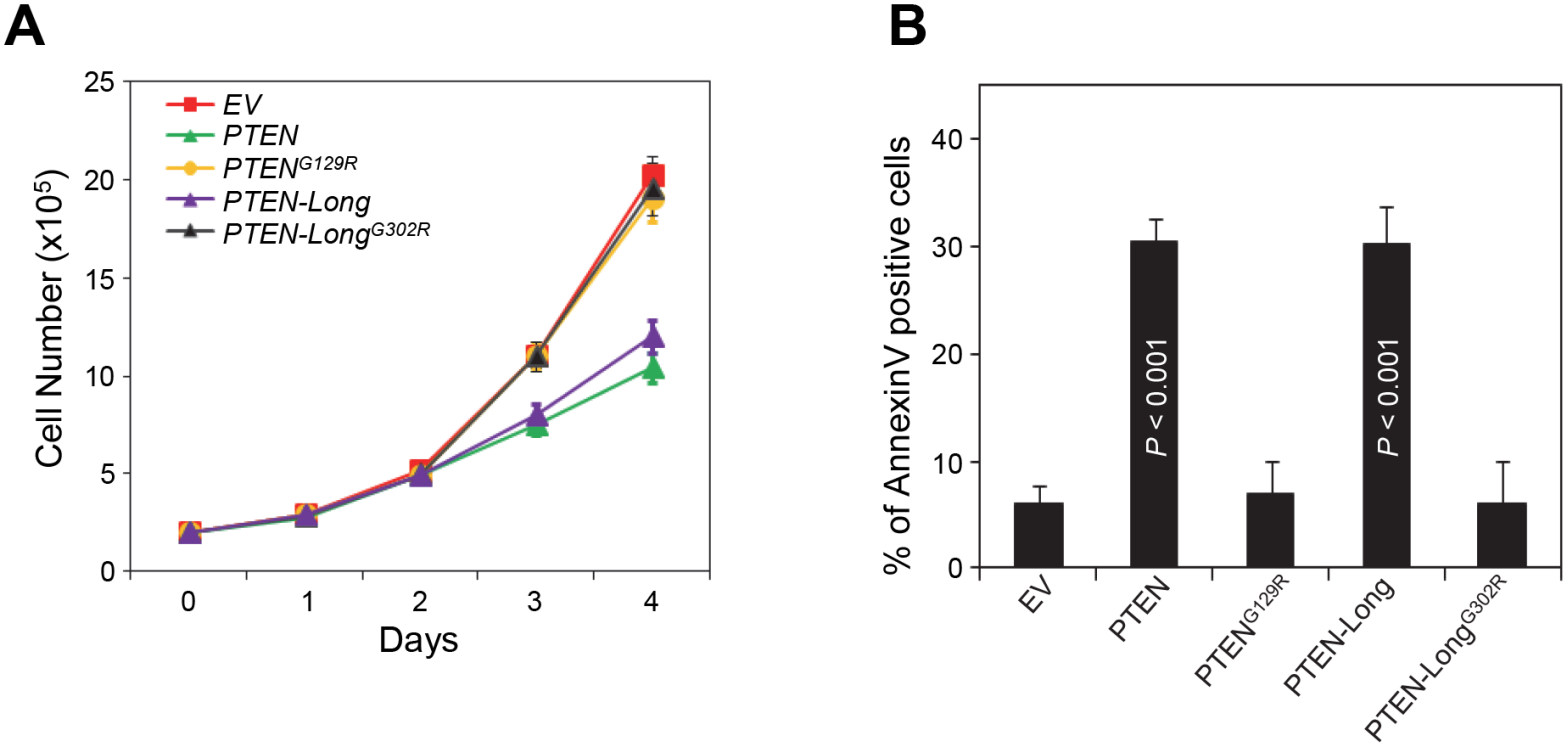
Overexpression of PTEN-Long inhibits cell proliferation and induces cell death. **A**, Cell proliferation assay indicating reduced growth in 786-0 cells expressing PTEN or PTEN-Long compared to PTEN^G129R^, PTEN-Long^G302R^, or empty vector transfected cells. **B**, AnnexinV staining followed by flow cytometry analysis indicating overexpression of PTEN or PTEN-Long promoted serum starvation-induced apoptosis.

PTEN has been reported to play a crucial role in modulating proliferation and apoptosis by reducing the levels of PIP3 that activates Akt, a central regulator of survival and death of cell. To understand the role of PTEN-Long in regulating cell proliferation and cell death, we stably overexpressed PTEN, PTEN^G129R^, PTEN-Long or PTEN-Long^G302R^ in 786-0 cells and compared their effects on cell growth and induction of cell death. We found, similar to PTEN, overexpression of PTEN-Long in 786-0 cells inhibited cell proliferation and this depends on its lipid phosphatase activity as PTEN-Long^G302R^ showed no effect (Fig. 3A).

To understand the role of PTEN-Long in serum starvation-induced cell death, 786-0 cells expressing different constructs as indicated were serum-starved for 24 h, 48 h and 72 h and then subjected to Annexin-V-FITC staining followed by flow cytometry analysis. As shown in Fig. 3B, a total of 72 h of serum starvation increased the number of apoptotic PTEN-Long-expressing cells by about 3-fold, as compared to vector-control cells, which is similar to PTEN-expressing cells (Fig. 3B). Moreover, the apoptotic effects of both PTEN and PTEN-Long are dependent on their lipid phosphatase activity, as neither PTEN^G129R^ nor PTEN-Long^G302R^ induced cell death.

### Overexpression of PTEN-Long inhibits cell migration and reduce cell invasion

**Figure 4:**
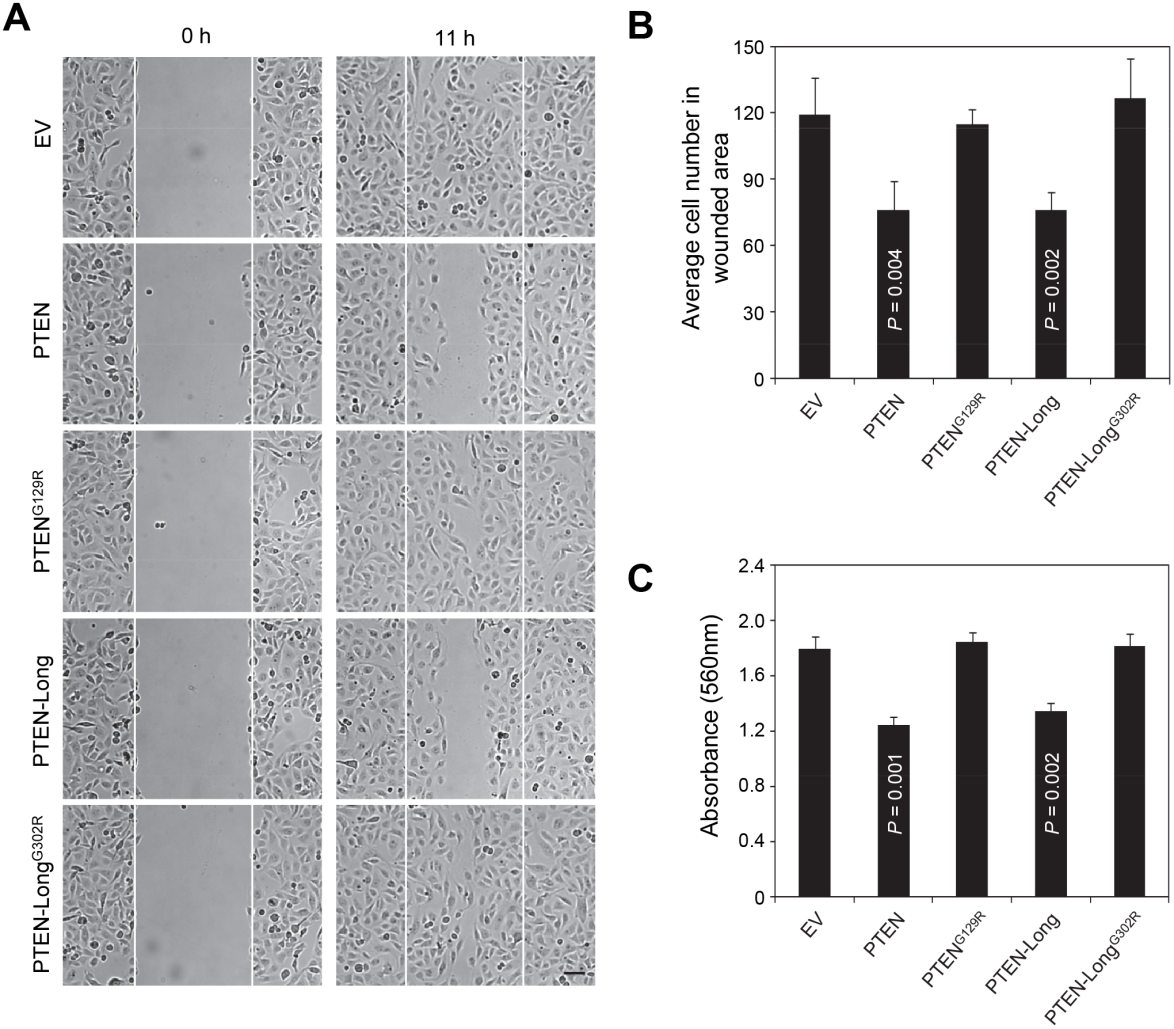
Overexpression of PTEN-Long inhibits cell migration and reduces cell invasion. **A**, 786-0 cells stably expressing indicated proteins were subjected to *in vitro* wounding and followed by phase-contrast imaging. The fields in between the white lines indicate the wound track. The scale bar represents 20 µm. **B**, The number of cells migrated in the wound track from 6 random separate fields were counted for each cell and expressed as a mean ± SD (error bars). **C**, Invasive properties of 786-0 cells were inhibited by 30% by PTEN overexpression and by 25% by PTEN-Long overexpression, as compared to empty vector control cells.

Next, we investigated the contribution of PTEN-Long to the migratory properties of 786-0 by scratch assay. First, we showed that the expression of PTEN inhibited the ability of 786-0 cells to migrate into a wound track, as compared with vector control cells (Fig. 4A, rows 1 and 2). Expression of PTEN-Long also inhibited migration of cells (Fig. 4A, rows 4) and this function was dependent on its lipid phosphatase activity, as both PTEN^G129R^ and PTEN-Long^G302R^ mutants failed to produce the same results (Fig. 4A, row 3 and 5). Quantitative analysis of cells migrated into wound area 11 h after scratching revealed a roughly 35% (*P*<0.001) inhibition in migration of 786-0 cells into the wound track by overexpression of PTEN-Long or PTEN (Fig. 4B). Because the G to R mutants of PTEN and PTEN-Long are still theoretically functional as protein phosphatase, failure of the G to R mutants to inhibit migration of 786-0 indicates that protein phosphatase activity of PTEN and PTEN-Long is not sufficient for this function.

To determine whether PTEN-Long also affects renal cancer cell invasion, we used a cytoselect 24-well cell invasion kit. 786-0 cells were transfected with different constructs individually, as indicated, and were subjected to an invasion assay. The results showed that PTEN-Long and PTEN expressing cells showed a roughly 25% and 30% decrease in cell invasion respectively, as compared with empty vector alone or PTEN-Long^G302R^ or PTENG^129R^ expressing cells (Fig. 4C).

### Purified PTEN-Long enters 786-0 cells, inhibits PI3K signal, and induces cell death

**Figure 5:**
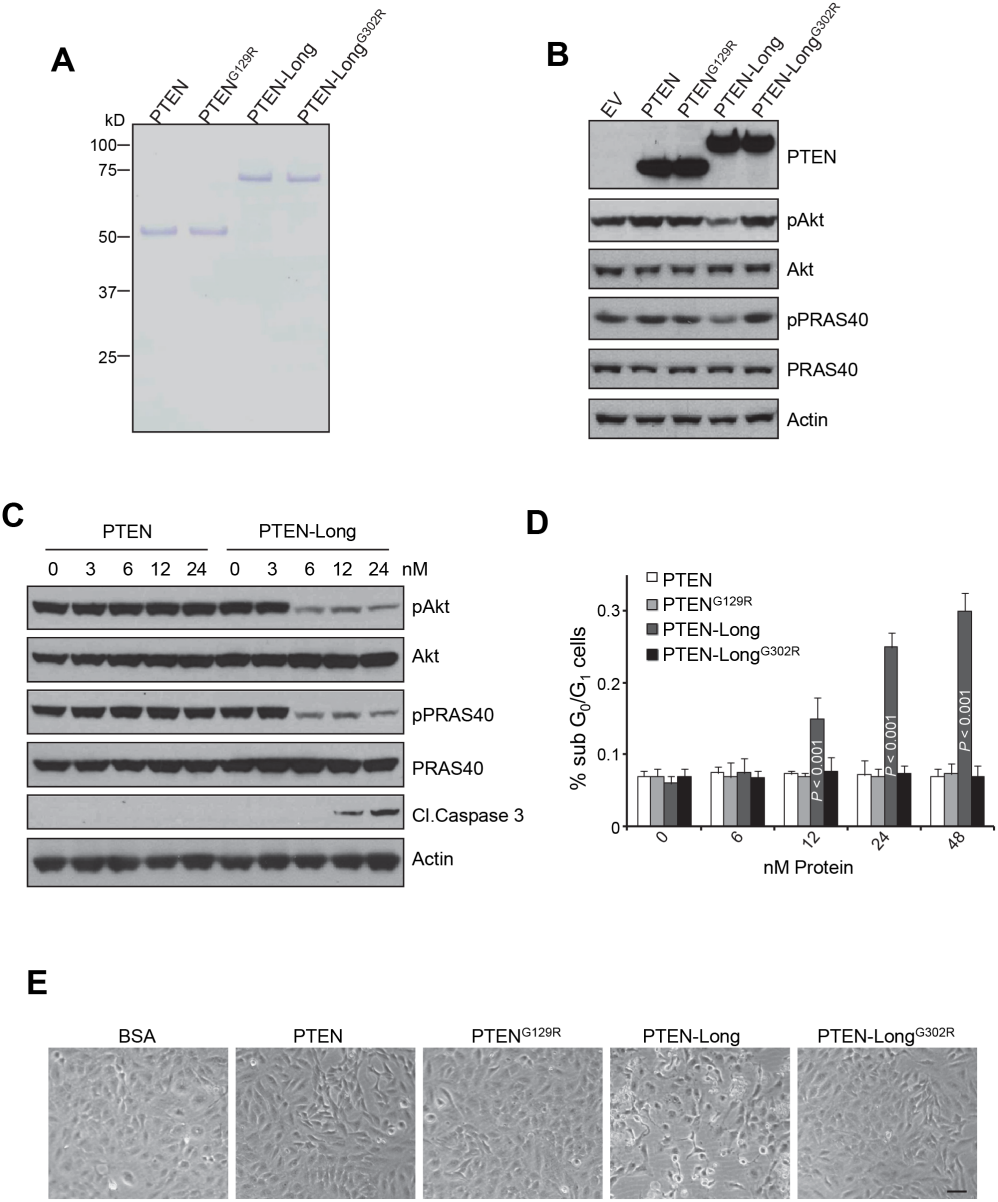
PTEN-Long treatment in culture inhibits PI3K signal and induces apoptosis in 786-0 cells. **A**, Coomassie stained gel showing representative protein preparations of PTEN, PTEN^G129R^, PTEN-Long, and PTEN-Long^G302R^. **B**, Effect of treatment with 25 nM PTEN-Long or other related proteins for 1h on pAkt and pPRAS40. PTEN-Long but not other proteins reduced intracellular phosphorylation of the Akt and PRAS40 in 786-0 cells. **C** and **D**, Effect of treatment with PTEN-Long or related proteins at different doses for 24 h on serum-starved 786-0 cells showed that PTEN-Long but not PTEN suppressed PI3K signaling and induced apoptosis, indicated by cleavage of caspase 3 in a dose-dependent manner. Percentages of apoptotic cells were also determined by PI/FACS (**C**). n = 4; bars represent the means ± SD. **E**, Representative pictures of the treated cells were taken by phase contrast photography. Protein concentrations were 50 nM. The scale bar represents 20 µm.

As PTEN-Long is a potential protein-type drug for cancer therapy [24], we next tested whether PTEN-Long is able to affect cellular signaling as an exogenous agent. Recombinant PTEN-Long, PTEN-Long^G302R^, PTEN and PTEN^G129R^ were expressed in bacteria and purified. The effects of proteins on cells were evaluated by direct application of purified products into cultured cells followed by Western blotting analysis of cell lysates prepared. The results showed that the only treatment of 786-0 cells in culture for 1 hr with purified PTEN-Long, but not other proteins, reduced intracellular phosphorylation of the Akt and PRAS40 in 786-0 cells (Fig. 5A and 5B). Moreover, dose-response experiments with 786-0 cells deprived of serum and treatment for 24 h showed that PTEN-Long both suppressed PI3K signaling, which was indicated by reduced levels of phosphorylated Akt and PRAS40, and also induced cell apoptosis, indicated by cleavage of caspase 3 in a dose-dependent manner (Fig. 5C) and increased percentages of sub G_0_/G_1_ population determined by PI/FACS (Fig. 5D). In comparison, no effect was seen from treatment of cells with purified PTEN at various doses (Fig. 5C–5E), suggesting PTEN-Long has therapeutic advantage over PTEN.

**Figure 6:**
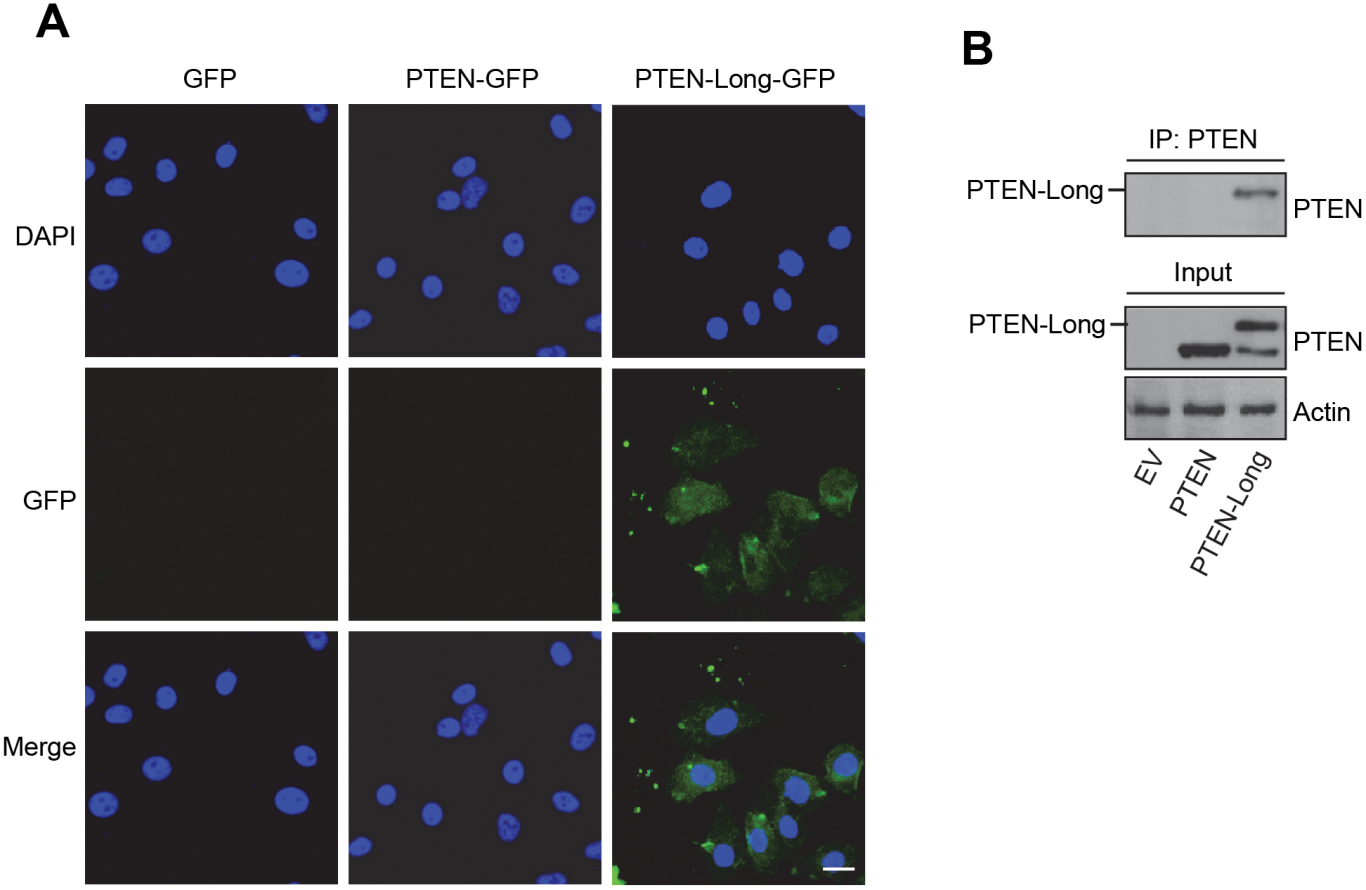
Entry of PTEN-Long into 786-0 cells. **A**, 786-0 cells treated with 100 nM purified PTEN-Long-GFP or PTEN-GFP for 5 h and imaged, showing PTEN-Long-GFP but not PTEN-GFP entered 786-0 cells. The scale bar represents 10 µm. **B**, Immunoprecipitation for PTEN on cell lysates pretreated with PTEN-Long or PTEN followed by Western blotting analysis with antibody against PTEN, showing only PTEN-Long was detected.

Previously, Parsons and colleagues reported an evolutionarily conserved poly-arginine stretch, similar to that in the cell-penetrating transactivator of transcription (TAT) protein from HIV, was present in the PTEN-Long N-terminal domain. This gives PTEN-Long the ability to enter cells. To test whether PTEN-Long entered 786-0 cells to execute killing function, we attached a green fluorescence protein (GFP) tag to the C-terminal of PTEN-Long and PTEN. 786-0 cells were treated with 100 nM purified PTEN-Long-GFP or PTEN-GFP for 5 h. Cells were washed, fixed, and imaged under fluorescence microscope. GFP signal was only detected from 786-0 cells pre-treated with PTEN-Long-GFP but not PTEN-GFP, indicating only PTEN-Long entered cells (Fig. 6A). To confirm this observation, we concentrated PTEN or PTEN-Long from cell lysate by immunoprecipitation with antibody against PTEN and analyzed by Western blotting, which showed only PTEN-Long was detected (Fig. 6B, upper panel).

### PTEN-Long inhibits the proliferation of 786-0 cells in xenograft mouse model

**Figure 7:**
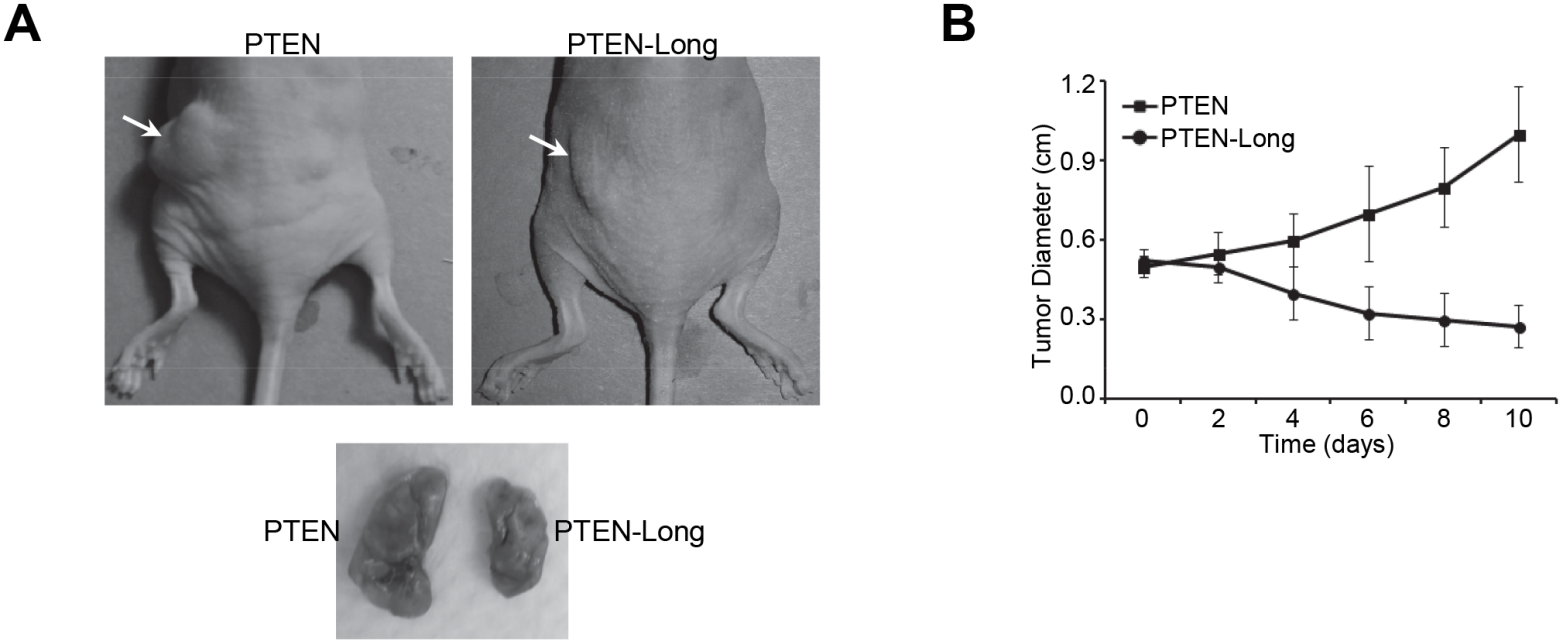
PTEN-Long inhibits the proliferation of 786-0 cells in xenograft mouse model. **A**, 786-0 cells engrafted nude mice were treated with 5 mg/kg of PTEN or PTEN-Long for 6 days. PTEN-Long but not PTEN inhibited tumor growth. **B**, Graph of tumor diameters as measured by calipers and treated with either PTEN or PTEN-Long at 5 mg/kg for different time periods. n = 6; bars represent the means ± SD.

We further explored the effect of PTEN-Long on proliferation of 786-0 cells in mouse xenograft model. We engraft 786-0 cells subcutaneously into athymic nude mice and waited until their tumor growth reached ∼0.5 cm of diameter. Mice carrying tumors were randomly divided into two groups and were injected intraperitoneally with PTEN or PTEN-Long at 5 mg/kg on daily basis. We found treatment with PTEN-Long caused tumor regression after 4 days (Fig. 7A and 7B). Taken together, these results indicated PTEN-Long protein is a potential protein drug against ccRCC.

## Discussion

RCC, like most cancers of the kidney, are exceptionally difficult to treat therapeutically, with only a small percentage of patients being cured [6]. Present treatment options, such as standard chemotherapy and biologic agents have not been effective and have many shortcomings [6]. In patients with metastatic RCC, systemic chemotherapy has produced a limited response [6]. With increased knowledge of RCC genetics and its molecular pathways, there are greater opportunities for truly targeted therapies. Targeting the PI3K-Akt-mTOR signal transduction pathway mutated in RCC has had promising results in recent studies, and in unselected patients with metastatic RCC, mTOR inhibitors have been shown to have clinical efficacy and low toxicity [6].

In this study, we show that protein expression of PTEN-Long is reduced or completely lost in ccRCCs at high frequency, suggesting it plays an important role in renal cell carcinogenesis. PTEN-Long is produced from an alternative translation start site 519 base pairs upstream of the ATG initiation sequence of PTEN, adding additional 173 amino acids to the N-terminal of PTEN [24], [26]. Like classical PTEN, PTEN-Long acts as an antagonist of the PI3K-Akt-mTOR pathway. Unlike PTEN, PTEN-Long is a member-permeable that is secreted from cells and capable to enter neighboring cells. After entering other cells, PTEN-Long dephosphorylates PIP3, antagonizes PI3K-Akt signaling, and induces cell death. Our study demonstrated that PTEN-Long played an important role in renal cell carcinogenesis and by providing a means to restore a functional tumor-suppressor protein to tumor cells, PTEN-Long has therapeutic potential.

Some limitations of this study need to be acknowledged. The nature of this study was retrospective and therefore only limited conclusions can be drawn. Although the therapeutic effect of PTEN-Long was tested in xenografted renal tumor mouse model, a prospective animal model should be established to prove loss of PTEN-Long leads to development or accelerated progression of RCC. Furthermore, it’s appealing to test if PTEN-Long and PTEN have synergistic effects. Does PTEN-Long have biochemical activity other than lipid or protein phosphatase reported for PTEN? Another challenge is to develop robust reproducible biomarkers that can be used in the clinic to identify patients most likely to benefit from PTEN-Long therapy. All these questions require broad and deep studies on PTEN-Long.
